# Genetic variation analysis and relationships among environmental strains of *Scedosporium apiospermum* sensu stricto in Bangkok, Thailand

**DOI:** 10.1371/journal.pone.0181083

**Published:** 2017-07-12

**Authors:** Thanwa Wongsuk, Potjaman Pumeesat, Natthanej Luplertlop

**Affiliations:** 1 Department of Microbiology and Immunology, Faculty of Tropical Medicine, Mahidol University, Bangkok, Thailand; 2 Department of Clinical Pathology, Faculty of Medicine, Vajira Hospital, Navamindradhiraj University, Bangkok, Thailand; 3 Department of Medical Technology, Faculty of Science and Technology, Bansomdejchaopraya Rajabhat University, Bangkok, Thailand; Brigham Young University, UNITED STATES

## Abstract

The *Scedosporium apiospermum* species complex is an emerging filamentous fungi that has been isolated from environment. It can cause a wide range of infections in both immunocompetent and immunocompromised individuals. We aimed to study the genetic variation and relationships between 48 strains of *S*. *apiospermum* sensu stricto isolated from soil in Bangkok, Thailand. For PCR, sequencing and phylogenetic analysis, we used the following genes: actin; calmodulin exons 3 and 4; the second largest subunit of the RNA polymerase II; ß-tubulin exon 2–4; manganese superoxide dismutase; internal transcribed spacer; transcription elongation factor 1α; and beta-tubulin exons 5 and 6. The present study is the first phylogenetic analysis of relationships among *S*. *apiospermum* sensu stricto in Thailand and South-east Asia. This result provides useful information for future epidemiological study and may be correlated to clinical manifestation.

## Introduction

The *Scedosporium apiospermum* species complex is a group of filamentous fungi that have been reported in cystic fibrosis (CF) patients [1]. It can be isolated from the environment, especially in human-impacted areas such as playgrounds, industrial and agricultural zones [2]. In Thailand, *S*. *apiospermum* has been reported in brain abscesses of near-drowning and renal transplant patients [3,4], and *S*. *boydii* infections have also been reported in brain tissue of renal transplant patient [5]. Additionally, two Swiss tourists who nearly drowned in the tsunami disaster in Thailand were found to be infected with *S*. *apiospermum* [6].

Several studies have tried to classify the genus *Scedosporium*. One previous study defined “*S*. *apiospermum* species complex” as comprising *S*. *apiospermum*, *S*. *boydii*, and *Pseudallescheria angusta* [7], but another study defined it as five species: *S*. *apiospermum* sensu stricto, *S*. *boydii*, *S*. *aurantiacum*, *S*. *dehoogii*, and *S*. *minutispora* [8]. The identification of *Scedosporium* at species level is important as epidemiology, clinical outcomes, and antifungal susceptibility are species-specific [9,10].

In order to overcome the difficulties in identifying *Scedosporium* species by routine microbiological methods, several molecular techniques have been proposed such as quantitative real-time PCR (qPCR), PCR-based reverse line blot (PCR-RLB), and loop-mediated isothermal amplification (LAMP) [11,12]. Additionally, globally standardized genotyping of *S*. *apiospermum* and *S*. *boydii*, the Multi-Locus Sequences Typing (MLST) scheme, was developed by Bernhardt *et al*. [13]. The MLST scheme amplifies sequences at five genetic loci–actin (ACT), calmodulin exons 3 and 4 (CAL), the second largest subunit of RNA polymerase II gene (RPB2), ß-tubulin exons 2–4 (BT2), and manganese superoxide dismutase (SOD2) and has been found to have strong repeatability [13–15]. The allele types (ATs) and sequences types (STs) numbers of the consensus MLST scheme can be obtained through the Fungal MLST Database (http://mlst.mycologylab.org/). In our previous study, we investigated the spatial distribution of the *S*. *apiospermum* species complex in soil in Bangkok, Thailand. We found that the *S*. *apiospermum* species complex is widespread in soil across Bangkok and detected predominance of *S*. *apiospermum* sensu stricto (72%) [16].

Here, we continue the study by considering the genetic variation and relationships among *S*. *apiospermum* sensu stricto isolated from soil in Bangkok. The data may be used for further epidemiological research, for which it is important to recognize different strains and subspecies.

## Materials and methods

### Strains

We used 48 isolates of *S*. *apiospermum* sensu stricto from our stock collection. Each isolate originated from soil and had previously been typed by PCR of the beta-tubulin gene (exons 5 and 6) [16]. Each isolate was labeled as TMMI (Department of Microbiology and Immunology, Faculty of Tropical Medicine, Mahidol University yeast and moulds culture collection) with a unique identification number (Table 1).

**Table 1:** GenBank accession numbers of sequences.

Number	Collection name	Isolate name	GenBank accession numbers							
			RPB2	TEF-1α	ITS	LSU	ACT	CAL	SOD2	BT2
1	TMMI001	A43A2D7	KY122727	KY122775	KY122823	KY122871	KY122919	KY122967	KY123015	KY123063
2	TMMI002	A54A2E9	KY122728	KY122776	KY122824	KY122872	KY122920	KY122968	KY123016	KY123064
3	TMMI003	A45A2D8	KY122729	KY122777	KY122825	KY122873	KY122921	KY122969	KY123017	KY123065
4	TMMI004	A103A3A6	KY122730	KY122778	KY122826	KY122874	KY122922	KY122970	KY123018	KY123066
5	TMMI005	A25A1F6	KY122731	KY122779	KY122827	KY122875	KY122923	KY122971	KY123019	KY123067
6	TMMI006	A51A2E1	KY122732	KY122780	KY122828	KY122876	KY122924	KY122972	KY123020	KY123068
7	TMMI009	A25A1F4	KY122733	KY122781	KY122829	KY122877	KY122925	KY122973	KY123021	KY123069
8	TMMI010	A93A2G7	KY122734	KY122782	KY122830	KY122878	KY122926	KY122974	KY123022	KY123070
9	TMMI011	A24B1D6	KY122735	KY122783	KY122831	KY122879	KY122927	KY122975	KY123023	KY123071
10	TMMI015	A22A1B9	KY122736	KY122784	KY122832	KY122880	KY122928	KY122976	KY123024	KY123072
11	TMMI016	A35A2C8	KY122737	KY122785	KY122833	KY122881	KY122929	KY122977	KY123025	KY123073
12	TMMI017	A101B1I9	KY122738	KY122786	KY122834	KY122882	KY122930	KY122978	KY123026	KY123074
13	TMMI019	A101A2I6	KY122739	KY122787	KY122835	KY122883	KY122931	KY122979	KY123027	KY123075
14	TMMI020	B61A1G8	KY122740	KY122788	KY122836	KY122884	KY122932	KY122980	KY123028	KY123076
15	TMMI023	B61B1G4	KY122741	KY122789	KY122837	KY122885	KY122933	KY122981	KY123029	KY123077
16	TMMI025	B21B1A9	KY122742	KY122790	KY122838	KY122886	KY122934	KY122982	KY123030	KY123078
17	TMMI028	B61B1G5	KY122743	KY122791	KY122839	KY122887	KY122935	KY122983	KY123031	KY123079
18	TMMI030	B92B4D4	KY122744	KY122792	KY122840	KY122888	KY122936	KY122984	KY123032	KY123080
19	TMMI031	B63B1I5	KY122745	KY122793	KY122841	KY122889	KY122937	KY122985	KY123033	KY123081
20	TMMI033	B33B1D1	KY122746	KY122794	KY122842	KY122890	KY122938	KY122986	KY123034	KY123082
21	TMMI034	B83B2C8	KY122747	KY122795	KY122843	KY122891	KY122939	KY122987	KY123035	KY123083
22	TMMI036	B64B1I9	KY122748	KY122796	KY122844	KY122892	KY122940	KY122988	KY123036	KY123084
23	TMMI037	B81B2C7	KY122749	KY122797	KY122845	KY122893	KY122941	KY122989	KY123037	KY123085
24	TMMI038	B32B1C7	KY122750	KY122798	KY122846	KY122894	KY122942	KY122990	KY123038	KY123086
25	TMMI039	B35B1F4	KY122751	KY122799	KY122847	KY122895	KY122943	KY122991	KY123039	KY123087
26	TMMI040	C92B2	KY122752	KY122800	KY122848	KY122896	KY122944	KY122992	KY123040	KY123088
27	TMMI042	C35I7	KY122753	KY122801	KY122849	KY122897	KY122945	KY122993	KY123041	KY123089
28	TMMI043	C24D3	KY122754	KY122802	KY122850	KY122898	KY122946	KY122994	KY123042	KY123090
29	TMMI044	C24D4	KY122755	KY122803	KY122851	KY122899	KY122947	KY122995	KY123043	KY123091
30	TMMI046	C32F3	KY122756	KY122804	KY122852	KY122900	KY122948	KY122996	KY123044	KY123092
31	TMMI047	C84G3	KY122757	KY122805	KY122853	KY122901	KY122949	KY122997	KY123045	KY123093
32	TMMI048	C61H8	KY122758	KY122806	KY122854	KY122902	KY122950	KY122998	KY123046	KY123094
33	TMMI050	C31E9	KY122759	KY122807	KY122855	KY122903	KY122951	KY122999	KY123047	KY123095
34	TMMI051	C25D8	KY122760	KY122808	KY122856	KY122904	KY122952	KY123000	KY123048	KY123096
35	TMMI052	C65F2	KY122761	KY122809	KY122857	KY122905	KY122953	KY123001	KY123049	KY123097
36	TMMI053	C84G2	KY122762	KY122810	KY122858	KY122906	KY122954	KY123002	KY123050	KY123098
37	TMMI054	C13B1	KY122763	KY122811	KY122859	KY122907	KY122955	KY123003	KY123051	KY123099
38	TMMI055	C95F3	KY122764	KY122812	KY122860	KY122908	KY122956	KY123004	KY123052	KY123100
39	TMMI057	D11B8	KY122765	KY122813	KY122861	KY122909	KY122957	KY123005	KY123053	KY123101
40	TMMI058	D32A8	KY122766	KY122814	KY122862	KY122910	KY122958	KY123006	KY123054	KY123102
41	TMMI060	D32A5	KY122767	KY122815	KY122863	KY122911	KY122959	KY123007	KY123055	KY123103
42	TMMI061	D11A1	KY122768	KY122816	KY122864	KY122912	KY122960	KY123008	KY123056	KY123104
43	TMMI062	D32A4	KY122769	KY122817	KY122865	KY122913	KY122961	KY123009	KY123057	KY123105
44	TMMI063	D35B9	KY122770	KY122818	KY122866	KY122914	KY122962	KY123010	KY123058	KY123106
45	TMMI067	G15G2C5	KY122771	KY122819	KY122867	KY122915	KY122963	KY123011	KY123059	KY123107
46	TMMI070	G92G3B6	KY122772	KY122820	KY122868	KY122916	KY122964	KY123012	KY123060	KY123108
47	TMMI071	G14G1H2	KY122773	KY122821	KY122869	KY122917	KY122965	KY123013	KY123061	KY123109
48	TMMI072	G12G1E3	KY122774	KY122822	KY122870	KY122918	KY122966	KY123014	KY123062	KY123110

### DNA extraction

DNA was extracted with an E.Z.N.A. Fungal DNA mini kit (Omega Bio-tek). The DNA samples were quantified and the quality was checked with a NanoDrop 2000 spectrophotometer (Thermo Fisher, Wilmington, DE, USA) and stored at −20°C until further use.

### Molecular biology technique

For PCR, sequencing and phylogenetic analysis we used eight genes: ACT, CAL, RPB2, BT2, SOD2, internal transcribed spacer (ITS), transcription elongation factor 1α (TEF-1α), and 28S large subunit ribosomal RNA (LSU). PCR amplification of eight gene regions was carried out with the specific primer pairs listed in Table 2. Each PCR reaction mixture was performed in 25-μl final volume containing: 0.5 μM of each primer, KAPA 2G Fast HS ReadyMix PCR kit with loading dye (KAPA Biosystems, USA), nuclease-free water and genomic DNA. PCR amplifications were carried out in a T100 Thermal Cycler (Bio-Rad) according to the following protocol: an initial step of 96°C for 6 min, followed by 35 cycles of 94°C for 1 min, an annealing temperature that specific with each gene (Table 2) for 1 min and 72°C for 45 s and a final extension step of 72°C for 10 min. Then, 5 μL of the PCR products were electrophoresed in a 1.5% agarose gel containing SERVA DNA Stain G (SERVA Electrophoresis GmbH, Germany) in 1X TBE buffer, and photographed using a Gel Doc XR+ system (Bio-Rad).

**Table 2:** List of primers used in the present study.

Gene	Primer name	Sequence primer (5′–3′)	Annealing temperature (°C)	Refs
ACT	ACT-1	TGGGACGATATGGAIAAIATCTTGCA	57	[13,17]
	ACT-4R	TCITCGTATTCTTGCTTIGAICTCCACAT		
CAL	CAL-FW	GACTATTCACTAACAACGCTGTG	55	[13]
	CAL-RW	GTCTAGTATAATCAAATCGTTAGAG		
RPB2	RPB2-5F	GAYGAYMGWGATCAYTTYGG	55	[13,18]
	RPB2-7R	CCCATRGCTTGYTTRCCCAT		
BT2	BT2a	GGTAACCAAATCGGTGCTGCTTTC	58	[13,19]
	BT2b	ACCCTCAGTGTAGTGACCCTTGGC		
SOD2	SOD2-F3	TCACCACGATAAACACCACC	55	[14]
	SOD2-R3	CGTCGATACCCAAGAGAGGA		
ITS	ITS5	GGAAGTAAAAGTCGTAACAAGG	55	[13]
	ITS4	TCCTCCGCTTATTGATATGC		
TEF-1α	EF1-983F	GCYCCYGGHCAYCGTGAYTTYAT	58	[20]
	EF1-1567R	ACHGTRCCRATACCACCRATCTT		
LSU	LROR	ACCCGCTGAACTTAAGC	55	[20]
	LR5	CCTGAGGGAAACTTCG		

Each PCR product was purified using a FavorPrep^TM^ GEL/PCR Purification Mini Kit (Favorgen Biotech Corporation, Taiwan) and sequenced using gene-specific both forward and reverse primers at the AITbiotech Pty Ltd (Singapore). The retrieved sequence files were analyses using BioEdit software (http://www.mbio.ncsu.edu/bioedit/bioedit.html) and compared with existing sequences in GenBank using BLASTn (http://blast.ncbi.nlm.nih.gov/Blast.cgi).

### Genotyping variation and relationship analysis of eight housekeeping genes

Nucleotide sequences of seven genes (ACT, CAL, RPB2, BT2, SOD2, ITS, TEF-1α) were used for genetic variation and relationship analysis together with ß-tubulin exons 5 and 6 gene (TUB). The following TUB sequences were downloaded from GenBank: KU533691, KU533702, KU533711, KU533712, KU533691, KU533656, KU533658, KU533655, KU533649, KU533673, KU533713, KU533674, KU533675, KU533695, KU533676, KU533703, KU533723, KU533704, KU533692, KU533693, KU533694, KU533677, KU533705, KU533678, KU533659, KU533679, KU533696, KU533680, KU533681, KU533682, KU533683, KU533707, KU533660, KU533684, KU533708, KU533706, KU533666, KU533697, KU533685, KU533650, KU533651, KU533652, KU533709, KU533710, KU533657, KU533716, KU533698, and KU533717. Each sequence was trimmed to the correct length with the start and the end of each gene (shown in Table 3). Genotype variation of each gene was analyzed by MLSTest v1.0.1.23 software, which was downloaded from http://ipe.unsa.edu.ar/software [21]. The ATs number was created by using the same software and allele profiles were used to assign the STs number. A phylogenetic network of each loci was performed with the neighbor-net algorithm by SplitsTree4 and downloaded from http://www.splitstree.org/ [22].

**Table 3:** Starting and ending sequences, number of alleles, number of polymorphisms, typing efficacy, and discrimination power of each gene.

Locus	Seq. start 5′–3′	Seq. end 5′–3′	No. of alleles	No. of polymorphisms	Typing efficacy	Discrimination power (95% confidence interval)
ACT	ATCAAC	GCGAAA	8	10	0.8	0.718 (0.627–0.809)
CAL	TTAAAG	TATCCC	8	9	0.889	0.791 (0.715–0.866)
RPB2	TAAGCT	TCCCAA	11	34	0.324	0.791 (0.7–0.881)
BT2	GACGAC	CAGTCC	12	41	0.293	0.825 (0.755–0.896)
SOD2	TCTCCA	GCGCGA	18	36	0.5	0.932 (0.905–0.959)
ITS	GGGATC	GACCTC	15	16	0.938	0.829 (0.745–0.913)
TEF	ATCAAG	TCTACA	6	6	1	0.727 (0.641–0.813)
TUB	GGCCAG	GCGAGC	8	19	0.421	0.688 (0.584–0.792)
Concatenated sequence	ATCAAC	GCGAGC	37	171	0.503	0.982 (0.965–1)

Sequences were concatenated by the following order respectively; ACT, CAL, RPB2, BT2, SOD2, ITS, TEF-1α and TUB. The best model of evolution for concatenated data set were selected from the Bayesian Information Criterion (BIC) in MEGA7 [23]. Model with the lowest BIC score was chosen to construct a maximum likelihood phylogenetic tree. A phylogenetic tree of the 48 aligned sequences excluding gaps and missing data for the heuristic search was obtained by applying the neighbor-joining method to a matrix of pairwise distances estimated using the maximum likelihood approach based on the best model in MEGA7 [23]. The tree is drawn to scale, with branch lengths measured in the number of substitutions per site. Codon positions included were 1st+2nd+3rd+Noncoding. There were a total of 4,841 positions in the final dataset. A bootstrap analysis was conducted with 1000 replications and bootstrap values ≥ 50% were shown above branches.

### Phylogenetic analysis of the concatenated sequences of ACT, CAL, RPB2, BT2 and SOD2 genes

To assess evolutionary relationships among isolates, all 48 generated concatenated of ACT, CAL, RPB2, BT2 and SOD2 genes sequences as well as 27 concatenated sequences of *S*. *apiospermum* sensu stricto (isolate IHEM 15555, IHEM 15553, IHEM 15552, IHEM 15551, IHEM 15148, IHEM 15146, IHEM 14764, IHEM 14763, IHEM 14762, IHEM 14465, IHEM 14463, IHEM 14462, IHEM 15151, IHEM 15149, IHEM 15643, IHEM 14276, IHEM 14275, IHEM 14273, IHEM 14270, IHEM 14269, IHEM 14268), BMU07462, BMU04729, BMU04111, BMU03882, BMU01117 and BMU00491) from other regions of the world and three *S*. *boydii* (isolate IHEM 14362, IHEM 14638 and IHEM 14457) concatenated sequences downloaded from GenBank were multiply-aligned using BioEdit. The concatenated sequences of *S*. *aurantiacum* isolate IHEM 15458, *S*. *angusta* isolate BMU01115, *Pseudallescheria fusoidea* isolate BMU01297 and *Pseudallescheria ellipsoidea* isolate BMU01118 were included in the phylogenetic analysis. GenBank accession numbers are listed in S1 Table.

MEGA7 [23] was used to selected the best model of evolution and a phylogenetic tree was constructed as described above. The analysis involved 82 nucleotides sequences. All position containing gaps and missing data were eliminated. There were a total of 2,958 positions in the final dataset. A bootstrap analysis was conducted with 1000 replications and bootstrap values ≥ 50% were shown above branches also.

### Nucleotide sequences accession numbers

The generated nucleotide sequences were deposited in GenBank under accession numbers and listed in Table 1.

## Results

PCR amplification of the eight genes was successful for all strains, with a single band investigated on gels after electrophoresis. The BLASTn algorithm was used for sequence similarity searching in the NCBI database. Sequence-based identities with a cutoff of ≥ 99% were considered significant [24,25].

Genotypic variation profiling of *S*. *apiospermum* sensu stricto was generated and showed 37 good different STs from 48 strains (Table 4). The numbers of ATs variation at each loci were 8 (ACT), 7 (CAL), 11 (RPB2), 12 (BT2), 18 (SOD2), 15 (ITS), 6 (TEF-1α), and 8 (TUB) (Table 5). The number of polymorphisms, typing efficacy, and power of discrimination (95% confidential interval) were calculated (Table 3).

**Table 4:** Allele types (ATs) and sequence types (STs) of ACT, CAL, RPB2, BT2, SOD2, ITS, TEF-1α, and TUB genes and STs.

Number	Collection name	Isolate name	ATs								STs
			ACT	CAL	RPB2	BT2	SOD2	ITS	TEF-1∞	TUB	
1	TMMI001	A43A2D7	1	1	1	1	1	1	1	1	1
2	TMMI002	A54A2E9	1	2	1	2	2	2	1	1	2
3	TMMI003	A45A2D8	2	3	2	3	3	3	2	1	3
4	TMMI004	A103A3A6	2	3	2	3	4	3	2	1	4
5	TMMI005	A25A1F6	3	4	3	4	5	4	3	2	5
6	TMMI006	A51A2E1	2	3	2	5	6	3	2	3	6
7	TMMI009	A25A1F4	3	4	3	4	7	4	3	2	7
8	TMMI010	A93A2G7	3	4	4	6	8	5	3	4	8
9	TMMI011	A24B1D6	2	3	2	6	4	3	2	5	9
10	TMMI015	A22A1B9	4	5	5	5	9	5	3	1	10
11	TMMI016	A35A2C8	2	3	2	6	6	3	2	5	11
12	TMMI017	A101B1I9	2	3	2	6	4	3	2	5	9
13	TMMI019	A101A2I6	1	2	1	1	10	2	1	1	12
14	TMMI020	B61A1G8	2	3	2	6	4	3	2	5	9
15	TMMI023	B61B1G4	1	2	1	2	2	2	4	1	13
16	TMMI025	B21B1A9	5	4	3	7	11	6	3	6	14
17	TMMI028	B61B1G5	1	2	1	2	2	2	4	1	13
18	TMMI030	B92B4D4	1	2	1	1	2	7	1	1	15
19	TMMI031	B63B1I5	1	2	1	2	2	2	4	1	13
20	TMMI033	B33B1D1	1	1	1	1	10	2	1	1	16
21	TMMI034	B83B2C8	2	3	2	6	12	8	2	5	17
22	TMMI036	B64B1I9	1	2	1	2	2	2	4	1	13
23	TMMI037	B81B2C7	2	3	2	6	13	3	2	5	18
24	TMMI038	B32B1C7	2	3	2	3	6	3	2	5	19
25	TMMI039	B35B1F4	2	3	2	6	4	3	2	5	9
26	TMMI040	C92B2	1	1	1	1	14	1	1	1	20
27	TMMI042	C35I7	2	3	2	6	4	3	2	5	9
28	TMMI043	C24D3	2	3	2	6	3	3	2	5	21
29	TMMI044	C24D4	2	3	2	6	13	9	2	5	22
30	TMMI046	C32F3	2	3	2	6	6	3	2	5	11
31	TMMI047	C84G3	1	1	6	1	2	2	1	1	23
32	TMMI048	C61H8	3	4	3	1	11	10	3	7	24
33	TMMI050	C31E9	2	3	2	6	13	3	2	5	18
34	TMMI051	C25D8	6	1	6	1	13	11	5	1	25
35	TMMI052	C65F2	1	1	6	2	10	2	1	1	26
36	TMMI053	C84G2	5	4	7	8	15	12	3	8	27
37	TMMI054	C13B1	1	2	6	1	10	2	5	1	28
38	TMMI055	C95F3	2	3	2	6	3	3	2	5	21
39	TMMI057	D11B8	3	4	8	6	7	4	3	4	29
40	TMMI058	D32A8	2	2	9	3	13	13	2	1	30
41	TMMI060	D32A5	7	4	4	6	16	4	3	4	31
42	TMMI061	D11A1	4	5	4	9	17	14	3	4	32
43	TMMI062	D32A4	2	3	2	3	3	3	2	1	3
44	TMMI063	D35B9	2	6	10	10	13	13	2	1	33
45	TMMI067	G15G2C5	3	5	8	11	16	5	3	3	34
46	TMMI070	G92G3B6	8	7	11	12	18	15	2	1	35
47	TMMI071	G14G1H2	1	8	6	1	14	7	1	1	36
48	TMMI072	G12G1E3	2	6	2	3	6	3	6	1	37

**Table 5:** Allele types number and frequency.

Gene	ATs	Frequency		Strain no.
ACT	1	14		TMMI001, TMMI002, TMMI019, TMMI023, TMMI028, TMMI030, TMMI031, TMMI033, TMMI036, TMMI040, TMMI047, TMMI052, TMMI054, TMMI071
	2	21		TMMI003, TMMI004, TMMI006, TMMI011, TMMI016, TMMI017, TMMI020, TMMI034, TMMI037, TMMI038, TMMI039, TMMI042, TMMI043, TMMI044, TMMI046, TTMI050, TMMI055, TMMI058, TMMI062, TMMI063, TMMI072
	3	6		TMMI005, TMMI009, TMMI010, TMMI048, TMMI057, TMMI067,
	4	2		TMMI015, TMMI061
	5	2		TMMI025, TMMI053
	6	1		TMMI051
	7	1		TMMI060
	8	1		TMMI070
CAL	1	6		TMMI001, TMMI033, TMMI040, TMMI047, TMMI051, TMMI052
	2	9		TMMI002, TMMI019, TMMI023, TMMI028, TMMI030, TMMI031, TMMI036, TMMI054, TMMI058
	3	18		TMMI003, TMMI004, TMMI006, TMMI011, TMMI016, TMMI017, TMMI020, TMMI034, TMMI037, TMMI038, TMMI039, TMMI042, TMMI043, TMMI044, TMMI046, TTMI050, TMMI055, TMMI062
	4	8		TMMI005, TMMI009, TMMI010, TMMI025, TMMI048, TMMI053, TMMI057, TMMI060
	5	3		TMMI015, TMMI061, TMMI067
	6	2		TMMI063, TMMI072
	7	1		TMMI070
RPB2	1	10		TMMI001, TMMI002, TMMI019, TMMI023, TMMI028, TMMI030, TMMI031, TMMI033, TMMI036, TMMI040
	2	19		TMMI003, TMMI004, TMMI006, TMMI011, TMMI016, TMMI017, TMMI020, TMMI034, TMMI037, TMMI038, TMMI039, TMMI042, TMMI043, TMMI044, TMMI046, TMMI050, TMMI055, TMMI062, TMMI072
	3	4		TMMI005, TMMI009, TMMI025, TMMI048
	4	3		TMMI010, TMMI060, TMMI061
	5	1		TMMI015
	6	5		TMMI047, TMMI051, TMMI052, TMMI054, TMMI071
	7	1		TMMI053
	8	2		TMMI057, TMMI067
	9	1		TMMI058
	10	1		TMMI063
	11	1		TMMI070
BT2	1	10		TMMI001, TMMI019, TMMI030, TMMI033, TMMI040, TMMI047, TMMI048, TMMI051, TMMI054, TMMI071
	2	6		TMMI002, TMMI023, TMMI028, TMMI031, TMMI036, TMMI052
	3	6		TMMI003, TMMI004, TMMI038, TMMI058, TMMI062, TMMI072
	4	2		TMMI005, TMMI009
	5	2		TMMI006, TMMI015
	6	16		TMMI010, TMMI011, TMMI016, TMMI017, TMMI020, TMMI034, TMMI037, TMMI039, TMMI042, TMMI043, TMMI044, TMMI046, TMMI050, TMMI055, TMMI057, TMMI060
	7	1		TMMI025
	8	1		TMMI053
	9	1		TMMI061
	10	1		TMMI063
	11	1		TMMI067
	12	1		TMMI070
SOD2	1		1	TMMI001	
	2		7	TMMI002, TMMI023, TMMI028, TMMI030, TMMI031, TMMI036, TMMI047	
	3		4	TMMI003, TMMI043, TMMI055, TMMI062	
	4		6	TMMI004, TMMI011, TMMI017, TMMI020, TMMI039, TMMI042	
	5		1	TMMI005	
	6		5	TMMI006, TMMI016, TMMI038, TMMI046, TMMI072	
	7		2	TMMI009, TMMI057	
	8		1	TMMI010	
	9		1	TMMI015	
	10		4	TMMI019, TMMI033, TMMI052, TMMI054	
	11		2	TMMI025, TMMI048	
	12		1	TMMI034	
	13		6	TMMI037, TMMI044, TMMI050, TMMI051, TMMI058, TMMI063	
	14		2	TMMI040, TMMI071	
	15		1	TMMI053	
	16		2	TMMI060, TMMI067	
	17		1	TMMI061	
	18		1	TMMI070	
ITS	1		2	TMMI001, TMMI040	
	2		10	TMMI002, TMMI019, TMMI023, TMMI028, TMMI031, TMMI033, TMMI036, TMMI047, TMMI052, TMMI054	
	3		17	TMMI003, TMMI004, TMMI006, TMMI011, TMMI016, TMMI017, TMMI020, TMMI037, TMMI038, TMMI039, TMMI042, TMMI043, TMMI046, TMMI050, TMMI055, TMMI062, TMMI072	
	4		4	TMMI005, TMMI009, TMMI057, TMMI060	
	5		3	TMMI010, TMMI015, TMMI067	
	6		1	TMMI025	
	7		2	TMMI030, TMMI071	
	8		1	TMMI034	
	9		1	TMMI044	
	10		1	TMMI048	
	11		1	TMMI051	
	12		1	TMMI053	
	13		2	TMMI058, TMMI063	
	14		1	TMMI061	
	15		1	TMMI070	
TEF-1α	1		9	TMMI001, TMMI002, TMMI019, TMMI030, TMMI033, TMMI040, TMMI047, TMMI052, TMMI071	
	2		21	TMMI003, TMMI004, TMMI006, TMMI011, TMMI016, TMMI017, TMMI020, TMMI034, TMMI037, TMMI038, TMMI039, TMMI042, TMMI043, TMMI044, TMMI046, TMMI050, TMMI055, TMMI058, TMMI062, TMMI063, TMMI070	
	3		11	TMMI005, TMMI009, TMMI010, TMMI015, TMMI025, TMMI048, TMMI053, TMMI057, TMMI060, TMMI061, TMMI067	
	4		4	TMMI023, TMMI028, TMMI031, TMMI036	
	5		2	TMMI051, TMMI054	
	6		1	TMMI072	
TUB	1		23	TMMI001, TMMI002, TMMI003, TMMI004, TMMI015, TMMI019, TMMI023, TMMI028, TMMI030, TMMI031, TMMI033, TMMI036, TMMI040, TMMI047, TMMI051, TMMI052, TMMI054, TMMI058, TMMI062, TMMI063, TMMI070, TMMI071, TMMI072	
	2		2	TMMI005, TMMI009	
	3		2	TMMI006, TMMI067	
	4		4	TMMI010, TMMI057, TMMI060, TMMI061	
	5		14	TMMI011, TMMI016, TMMI017, TMMI020, TMMI034, TMMI037, TMMI038, TMMI039, TMMI042, TMMI043, TMMI044, TMMI046, TMMI050, TMMI055	
	6		1	TMMI025	
	7		1	TMMI048	
	8		1	TMMI053	

**Fig 1:**
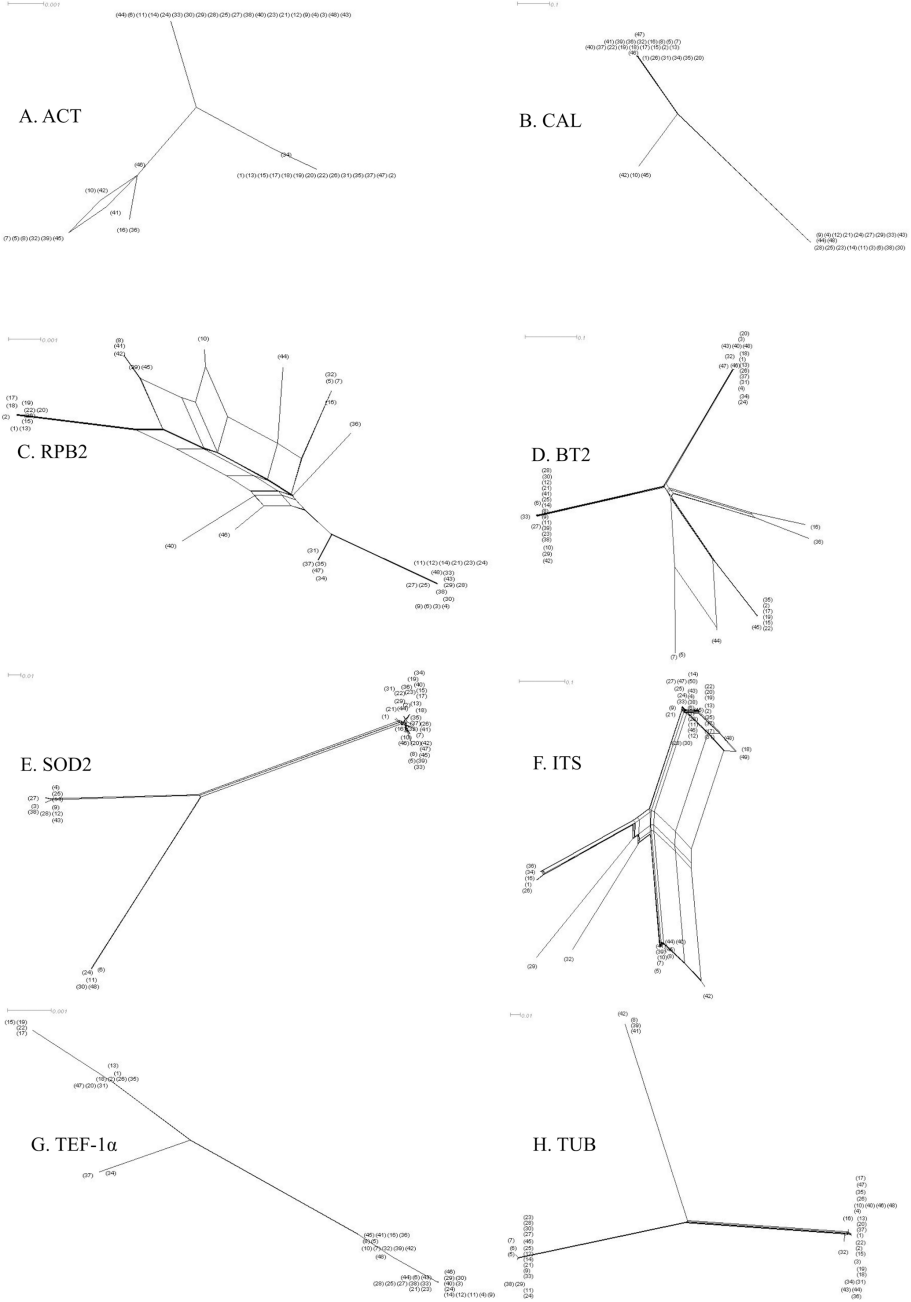
**Phylogenetic network (A–H).** SplitTree decomposition analysis using the neighbor-net algorithm of each of the eight genes i.e. A. ACT, B. CAL, C. RPB2, D. BT2, E. SOD2, F. ITS, G. TEF-1α and H. TUB (in blanket to show the number of the collection name of each strain).

**Fig 2:**
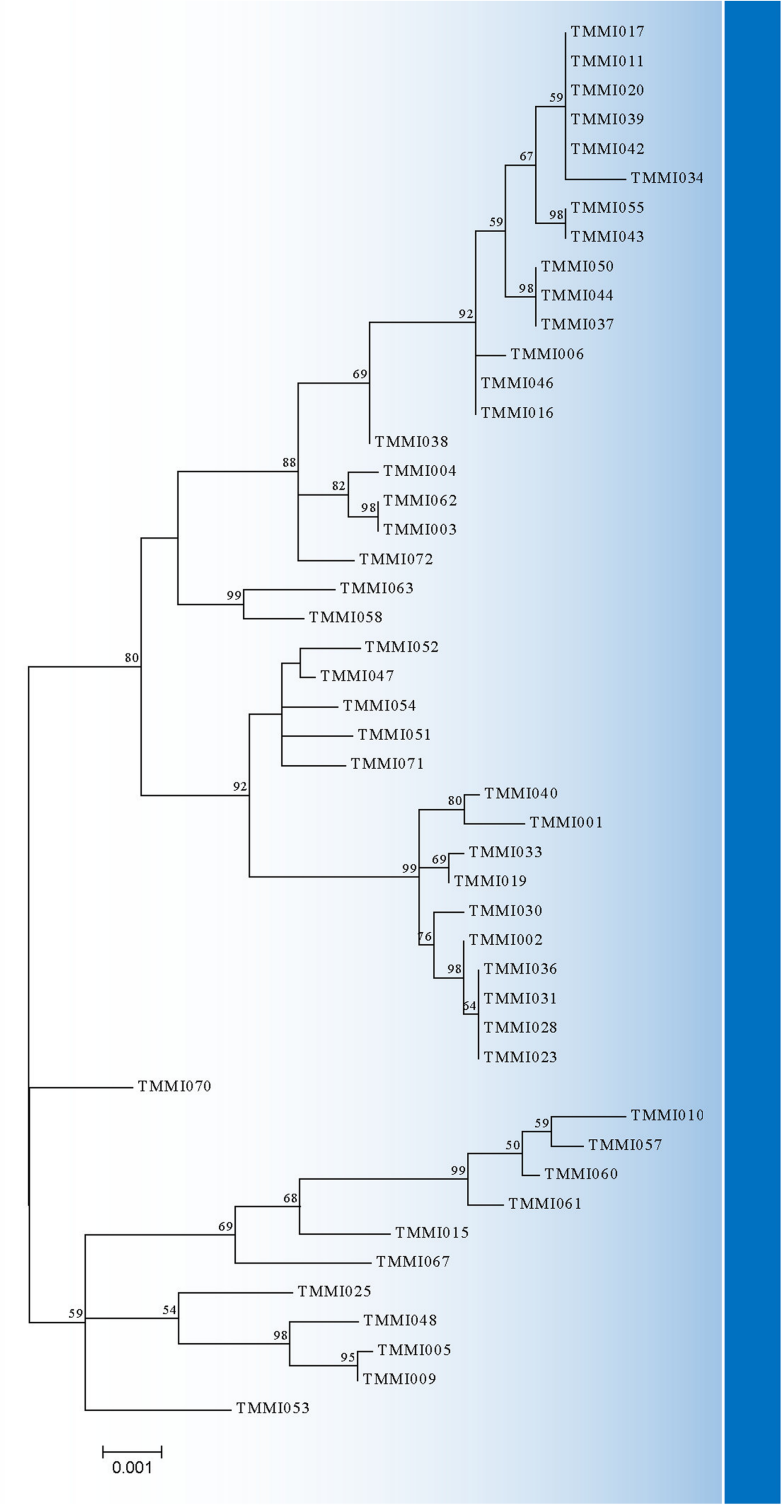
Molecular phylogenetic maximum likelihood analysis of the concatenated sequences of ACT, CAL, RPB2, BT2, SOD2, ITS, TEF-1α, and TUB genes. The tree with the highest log likelihood (-8390.7037) is shown.

We found diverse genetic relationships among the genotyped variants of 48 *S*. *apiospermum* sensu stricto after analyzing each gene with a neighbor-net algorithm (Fig 1). The best model for concatenated data set (ACT, CAL, RPB2, BT2, SOD2, ITS, TEF-1α and TUB, respectively) analyses was HKY+G+I (HKY: Hasegawa-Kishino-Yano; +G: Gamma distribution; +I: invariable sites) and the BIC score was 18004.65987. Therefore, the maximum likelihood phylogenetic tree of concatenated data set was created based on the HKY model [26]. A discrete Gamma distribution was used to model evolutionary rate differences among sites (5 categories (+G, parameter = 0.0500). The rate variation model allowed for some sites to be evolutionarily invariable ([+I], 48.7606% sites) (Fig 2).

**Fig 3:**
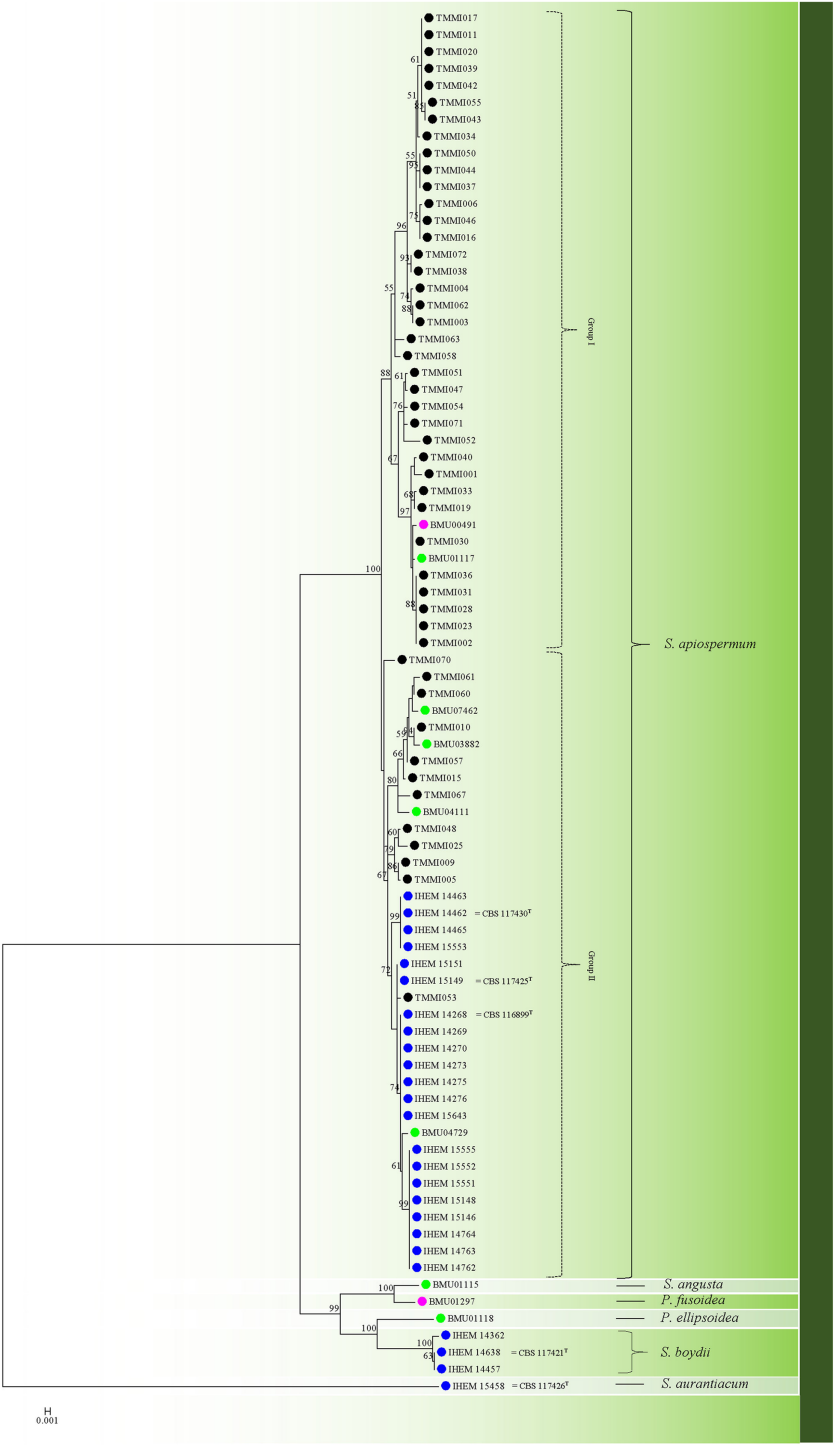
Molecular phylogenetic maximum likelihood analysis of the concatenated sequences of ACT, CAL, RPB2, BT2 and SOD2 genes. The tree with the highest log likelihood (-1105.3764) is shown. The black circle represents the strains from Thailand (environmental isolates), the blue circle represents the strains from France (clinical isolates), the green circle represents the strains from China (clinical isolates) and the pink circle represents the strains from Japan (clinical isolates).

To compare the genetic relationship of strains in Thailand with other regions of the world, the ACT, CAL, RPB2, BT2 and SOD2 sequences from previous studies in France, China and Japan were downloaded from GenBank. Data for these five genes were concatenated. TN93+G+I model (TN93: Tamura-Nei) was chosen according the best model of evolution analyses (the BIC score was 17282.89945). Then, a phylogenetic tree constructed by maximum likelihood analysis based on the TN93 model [27]. A discrete Gamma distribution was used to model evolutionary rate differences among sites (5 categories (+G, parameter = 0.1587)). The rate variation model allowed for some sites to be evolutionarily invariable ([+I], 42.7147% sites). As a tree (Fig 3), 82 nucleotide sequences comprised 2,958 positions were involved. *S*. *apiospermum* sensu stricto was strongly clustered together among Thai, French, Chinese and Japanese isolates (strongly supported by bootstrap value of 100%) and were subdivided into two groups (Group I and Group II).

## Discussion

The *Scedosporium apiospermum* species complex contains important opportunistic species. Giraud and Bouchara [8] and the European Confederation of Medical Mycology (ECMM)/International Society for Human and Animal Mycology (ISHAM) classify the novel nomenclature of the *S*. *apiospermum* species complex as comprising five species: *S*. *apiospermum* sensu stricto, *S*. *boydii* (= *Pseudallescheria boydii*), *S*. *aurantiacum*, *S*. *dehoogii*, and *S*. *minutispora*. In contrast, a recent study [7] defined the *S*. *apiospermum* species complex as only *S*. *apiospermum*, *S*. *boydii*, and *S*. *angusta* (= *Pseudalleschelia angusta*) because phylogenetic analysis of β-tubulin (BT2), γ-actin, transcriptional elongation factor 1α (TEF-1α), and internal transcribed spacer of the small ribosomal protein 60sS L10 (L1) distinguished *S*. *minutispora*, *S*. *aurantiacum*, and *S*. *dehoogii* from these three species. These days, there are numerous molecular techniques to determine genetic diversity that are robust and reproducible. MLST has been proposed as one of the best tools for genotypic and evolutionary studies.

In the fungal research field, four working groups have established public MLST schemes: (1) ISHAM established an MLST scheme for *Cryptococcus gattii*, *Cryptococcus neoformans*, *S*. *apiospermum*, *S*. *boydii*, *S*. *aurantiacum*, *Bipolaris australiensis*, *Bipolaris*. *hawaiiensis*, *Bipolaris spicifera* (http://mlst.mycologylab.org/); (2) Westerdijk Fungal Biodiversity Institute (CBS-KNAW Fungal Biodiversity Centre) established an MLST scheme for *Fusarium* spp. (http://www.westerdijkinstitute.nl/fusarium/); (3) Imperial College established an MLST scheme for *Candida albicans* and *Candida glabrata* (http://www.mlst.net/); and (4) Oxford University established an MLST scheme for *Aspergillus fumigatus*, *Pichia kudriavzevii* (*Candida krusei*) and *Candida tropicalis* (http://pubmlst.org/). MLST is clearly a powerful method for typing and studying genetic variation in microorganisms.

In this study, we used the MLST tool to study *S*. *apiospermum* sensu stricto previously isolated from soil samples. We chose eight housekeeping gene loci (ACT, BT2, CAL, ITS, RPB2, SOD2, TEF-1α, LSU) coupled with the TUB gene from our previous study to analyze the genetic variation and relationships among *S*. *apiospermum* sensu stricto strains, which are currently unknown and have not been previously analyzed genetically in Thailand or South-east Asia. We successfully sequenced all selected loci, but only the LSU sequences showed no polymorphisms (data not show). We identified 37 STs after combining the 8 genes (except LSU). In each gene fragment, the number of alleles varied; 8, 12, 8, 15, 11, 18, 6, 8 for ACT, BT2, CAL, ITS, RPB2, SOD2, TEF-1α, and TUB, respectively. The number of polymorphisms for each gene fragment varied from 6 (TEF-1α) to 41 (BT2). The sequences of the 48 *S*. *apiospermum* sensu stricto strains were accessed by a neighbor-net algorithm in SplitsTree4. SplitsTree4 graphs of ACT, CAL, RPB2, BT2, SOD2, ITS, TEF-1α, and TUB sequence data showed parallelograms that implied the character is shared by a set of species.

In terms of discriminatory power (DP) evaluation, SOD2 showed the highest DP (0.932); TUB showed the lowest DP, which was 0.688. Additionally, there were 37 STs, which could be grouped; each isolate had one ST type except ST3, ST9, ST11, ST13, ST18, and ST21, which had 2, 5, 2, 4, 2, and 2 ST types, respectively. In our study, SOD2 provided the highest number of alleles (18), and another study showed a similar high number of alleles for SOD2 [13]. Moreover, we combined five loci (ACT, CAL, RPB2, BT2 and SOD2) for objective genetic relationship analysis between Thailand (representing South-east Asia), China and Japan (Asia), and France (Europe): (i) to assess the relationship between clinical and environmental strains of *S*. *apiospermum*; and (ii) to assess the global variation between *S*. *apiospermum* strains. Our results detected a close relationship between the environmental strains from Thailand and the clinical strains from France [15], China and Japan [28]. Therefore, French, Chinese and Japan isolates originated in Thailand also. These data suggest that *S*. *apiospermum* sensu stricto isolates retrieved from different regions and different countries shared a close genetic relatedness. One limitation of our study was a lack of clinical isolates from Thailand to compare with our environmental strains. We hope that our data may be useful for other researchers in future study. Interestingly, TEF-1α used as a marker in an MLST scheme for *S*. *aurantiacum* (http://mlst.mycologylab.org/) including other filamentous fungi such as *Fusarium* [29], did not use *S*. *apiospermum* or *S*. *boydii*. In our data, TEF-1α presented the lowest number of alleles (6) and the DP was also quite low (0.688). This result may explain why TEF-1α was not used for *S*. *apiospermum* or *S*. *boydii*.

In summary, we here present the first phylogenetic analysis of relationships among *S*. *apiospermum* sensu stricto in Thailand and the South-east Asian region. The results provide valuable knowledge to assist future study and perhaps link the relationships of species in clinical settings.

## Supporting information

S1 Table Strains, specimens, countries and GenBank accession numbers of 34 sequences. (All sequences were using for phylogenetic analysis of the concatenated sequences of ACT, CAL, RPB2, BT2 and SOD2.)
(DOCX)
