# Supplementary material for: Genetic variation analysis and relationships among environmental strains of Scedosporium apiospermum sensu stricto in Bangkok, Thailand
Source: PLoS One. 2017 Jul 12;12(7):e0181083. doi: 10.1371/journal.pone.0181083 (PMC5507518; doi:10.1371/journal.pone.0181083)
Supplement: S1 Table — (All sequences were using for phylogenetic analysis of the concatenated sequences of ACT, CAL, RPB2, BT2 and SOD2.) (DOCX) [file pone.0181083.s001.docx]

**S1 Table. Strains, specimens, countries and GenBank accession numbers of 34 sequences.** (All sequences were using for phylogenetic analysis of the concatenated sequences of ACT, CAL, RPB2, BT2 and SOD2.)

|  | ***Organisms*** | **Strains** | **Specimen, Country** | **ACT** | **CAL** | **RPB2** | **BT2** | **SOD2** |
| --- | --- | --- | --- | --- | --- | --- | --- | --- |
| 1 | *S. apiospermum* | BMU00491 | Unknown, Japan | KP981108 | KP981131 | KP981179 | KP981214 | KP981154 |
| 2 | *S. apiospermum* | BMU01117 | Eye ball, China | KP981114 | KP981138 | KP981180 | KP981205 | KP981160 |
| 3 | *S. apiospermum* | BMU03882 | Sputum, China | KP981119 | KP981142 | KP981176 | KP981207 | KP981165 |
| 4 | *S. apiospermum* | BMU04111 | Joint fluid, China | KP981120 | KP981143 | KP981178 | KP981219 | KP981166 |
| 5 | *S. apiospermum* | BMU04729 | BALF, China | KP981121 | KP981144 | KP981198 | KP981216 | KP981167 |
| 6 | *S. apiospermum* | BMU07462 | CSF, China | KP981129 | KP981152 | KP981177 | KP981210 | KP981175 |
| 7 | *S. apiospermum* | IHEM 14268  (CBS 116899^T^) | Sputum, France | KT353225 | KT353286 | KT353347 | KT353469 | KT353408 |
| 8 | *S. apiospermum* | IHEM 14269 | Sputum, France | KT353226 | KT353287 | KT353348 | KT353470 | KT353409 |
| 9 | *S. apiospermum* | IHEM 14270 | Sputum, France | KT353227 | KT353288 | KT353349 | KT353471 | KT353410 |
| 10 | *S. apiospermum* | IHEM 14273 | Sputum, France | KT353228 | KT353289 | KT353350 | KT353472 | KT353411 |
| 11 | *S. apiospermum* | IHEM 14275 | Sputum, France | KT353229 | KT353290 | KT353351 | KT353473 | KT353412 |
| 12 | *S. apiospermum* | IHEM 14276 | Sputum, France | KT353230 | KT353291 | KT353352 | KT353474 | KT353413 |
| 13 | *S. apiospermum* | IHEM 15643 | Sputum, France | KT353231 | KT353292 | KT353353 | KT353475 | KT353414 |
| 14 | *S. apiospermum* | IHEM 15149 | Sputum, France | KT353232 | KT353293 | KT353354 | KT353476 | KT353415 |
| 15 | *S. apiospermum* | IHEM 15151  (CBS 117425^T^) | Sputum, France | KT353233 | KT353294 | KT353355 | KT353477 | KT353416 |
| 16 | *S. apiospermum* | IHEM 14462  (CBS 117430^T^) | Sputum, France | KT353240 | KT353301 | KT353362 | KT353480 | KT353423 |
| 17 | *S. apiospermum* | IHEM 14463 | Sputum, France | KT353241 | KT353302 | KT353363 | KT35348 | KT353424 |
| 18 | *S. apiospermum* | IHEM 14465 | Sputum, France | KT353242 | KT353303 | KT353364 | KT353482 | KT353425 |
| 19 | *S. apiospermum* | IHEM 14762 | Sputum, France | KT353243 | KT353304 | KT353365 | KT353483 | KT353426 |
| 20 | *S. apiospermum* | IHEM 14763 | Sputum, France | KT353244 | KT353305 | KT353366 | KT353484 | KT353427 |
| 21 | *S. apiospermum* | IHEM 14764 | Sputum, France | KT353245 | KT353306 | KT353367 | KT353485 | KT353428 |
| 22 | *S. apiospermum* | IHEM 15146 | Sputum, France | KT353246 | KT353307 | KT353368 | KT353486 | KT353429 |
| 23 | *S. apiospermum* | IHEM 15148 | Sputum, France | KT353247 | KT353308 | KT353369 | KT353487 | KT353430 |
| 24 | *S. apiospermum* | IHEM 15551 | Sputum, France | KT353248 | KT353309 | KT353370 | KT353488 | KT353431 |
| 25 | *S. apiospermum* | IHEM 15552 | Sputum, France | KT353249 | KT353310 | KT353371 | KT353489 | KT353432 |
| 26 | *S. apiospermum* | IHEM 15553 | Sputum, France | KT353250 | KT353311 | KT353372 | KT353490 | KT353433 |
| 27 | *S. apiospermum* | IHEM 15555 | Sputum, France | KT353251 | KP981115 | KT353373 | KT353491 | KT353434 |
| 28 | *S. boydii* | IHEM 14362 | Sputum, France | KT353256 | KT353317 | KT353378 | KT353496 | KT353439 |
|  | **Organisms** | **Strains** | **Specimen, Country** | **ACT** | **CAL** | **RPB2** | **BT2** | **SOD2** |
| 29 | *S. boydii* | IHEM 14638  (CBS 117421^T^) | Sputum, France | KT353202 | KT353263 | KT353324 | KT353446 | KT353385 |
| 30 | *S. boydii* | IHEM 14457 | Sputum, France | KT353201 | KT353262 | KT353323 | KT353445 | KT353384 |
| 31 | *S aurantiacum* | IHEM 15458  (CBS 117426^T^) | Sputum, France | KT353234 | KT353295 | KT353356 | JQ690970 | KT353417 |
| 32 | *S. angusta* | BMU01115 | Unknown, China | KP981112 | KP981135 | KP981194 | KP981200 | KP981158 |
| 33 | *P. fusoidea* | BMU01297 | Unknown, Japan | KP981117 | KP981140 | KP981195 | KP981221 | KP981163 |
| 34 | *P. ellipsoidea* | BMU01118 | Unknown, China | KP981115 | KP981137 | KP981190 | KP981215 | KP981161 |
